# Representing Physician Suicide Claims as Nanopublications: Proof-of-Concept Study Creating Claim Networks

**DOI:** 10.2196/34979

**Published:** 2022-07-01

**Authors:** Tiffany I Leung, Tobias Kuhn, Michel Dumontier

**Affiliations:** 1 Care and Public Health Research Institute Maastricht University Maastricht Netherlands; 2 Department of Internal Medicine (adjunct) Southern Illinois University School of Medicine Springfield, IL United States; 3 Department of Computer Science Vrije Universiteit Amsterdam Amsterdam Netherlands; 4 Institute of Data Science Maastricht Netherlands

**Keywords:** physician suicide, suicide, suicide prevention, physician well-being, physician mental health, nanopublication, physician, doctor, mental health, semantic publishing, bibliometrics, claim network, information distortion, misinformation

## Abstract

**Background:**

In the poorly studied field of physician suicide, various factors can contribute to misinformation or information distortion, which in turn can influence evidence-based policies and prevention of suicide in this unique population.

**Objective:**

The aim of this paper is to use nanopublications as a scientific publishing approach to establish a citation network of claims in peer-reviewed publications about the rate of suicide among US physicians.

**Methods:**

A list of articles from a previously published scoping literature review on physician suicide was used to identify those articles that commented on or investigated suicidal behaviors of physician populations, including students, postgraduate trainees, and practicing physicians. The included articles were from peer-reviewed publications and asserted a claim about the annual rate of physician suicide. Manual data extraction was performed to collect article (or resource) type, title, authors, digital object identifier or URI, publication year, claim (about annual physician suicide rate), data of last access of the article (eg, for a webpage), and citations supporting the claim. Additional articles, websites, or other links were only added to the set of claims if they were cited by a peer-reviewed article already included in the data set. A nanopublication was created for each article or resource using Nanobench with an investigator-developed literature-based claim nanopublication template.

**Results:**

A set of 49 claims concerning the rate of US physician suicide was represented as nanopublications. Analysis of the claim network revealed that (1) the network is not fully connected, (2) no single primary source of the claim could be identified, and (3) all end-point citations had a claim with no further citation, had no apparent claim, or could not be accessed to verify the claim. The nanopublication strategy also enabled the capture of variant claims published on a website.

**Conclusions:**

Nanopublications remain to be adopted in broader scientific publishing in medicine, especially in publishing about physician mental health and suicide. This proof-of-concept study highlights an opportunity for more coordinated research efforts in the subject of physician suicide. Our work integrates these various claims and enables the verification of nonauthoritative assertions, thereby better equipping researchers to advance evidence-based knowledge and to make informed statements in the advocacy of physician suicide prevention. Representing physician suicide rate claims as nanopublications can be extended and improved in future work.

## Introduction

Nanopublications are “core scientific statements with associated context” [1]. That is, scientific findings can be published as minimal pieces for computer interpretation, enabling nanopublications to cite other nanopublications unambiguously and reliably [2]. Furthermore, they are self-contained in that they contain scientific assertions as well as their provenance information and metadata; nanopublications can then be given reliable URIs for verification of the digital artifact and its entire reference tree [2]. The infrastructure allows the creation of citation, claim, and argumentation networks in which scientific statements are identified, connected, and verified [1].

In application, the use of nanopublications to represent scientific assertions in biomedical literature is not new. For example, the genetic basis for disease pathophysiology from DisGeNET has been mapped as nanopublication [3,4]. An Alzheimer disease research network built a web research community that organized research findings in an annotated knowledge base [5]. Applications largely involve data sets from the life science domains, including data on diseases, genes, proteins, drugs, and biological pathways [6].

In the field of physician suicide, disparate research, opinion, and position statements have been published in scholarly literature, with more than 60% of such literature published in the last 20 years alone [7]. Physician suicide has been reported in at least 37 countries, and many risk factors for suicidal behavior that affect the general population, such as inadequately diagnosed or treated mental health disorders or substance use disorders, also apply to physicians. More controversially, various unique risk factors have been suggested, including specialized knowledge of human physiology, easier access to lethal means of self-harm, personality traits selected in the physician training pathway, specialty of practice, and legal or licensing issues unique to the medical field [7].

Physician suicide is a serious issue for the medical workforce, globally and maximally leveraging available evidence toward prevention. Yet, even foundational information about the incidence of physician suicide remains poorly understood. In previous work, a claim network was manually constructed to trace the provenance of an often-cited claim that 300 to 400 US physicians die by suicide annually, which suggested that claim distortion and propagation of such misinformation about physician suicide incidence occurs in published literature [8]. This work drew from previous work on micropublications, which are a semantic model for scientific claims and evidence, which enables knowledge discovery and inference across networks of information [9,10]. A similar approach to identify and trace citation distortion had previously been carried out regarding a specific scientific claim about Alzheimer disease [11]. This paper extends this work by applying the nanopublication schema to the same physician suicide claim.

As literature about physician suicide is growing in parallel with the growth of scientific literature overall, which offers a unique opportunity to begin building core infrastructure to facilitate community learning, in a verifiable manner, about physician suicide. Such learning, founded on verifiability and reliability of available data, could support the needed vigilance of researchers, advocates, policy makers, and medical community in overcoming misinformation and information distortion about physician suicide.

The aim of this study is to use nanopublications as a scientific publishing approach to create a citation network of claims in peer-reviewed publications about the rate of suicide among US physicians. This is a proof-of-concept study for applying semantic web infrastructure to physician suicide research. To our knowledge, no such application to this field has previously been carried out. Facilitating the integration, interoperability, and findability of high-quality research on physician suicide would benefit evidence-based policies and interventions in suicide prevention among physicians.

## Methods

### Data Sources

A previous scoping review of the literature about physician suicide identified articles that commented on or investigated suicidal behaviors of physician populations, including students, postgraduate trainees, and practicing physicians [7]. Briefly, in that literature review, a medical librarian assisted in refining the research question, developing the search strategy, and conducting a search of relevant electronic databases, including Ovid Medline, PsycINFO, and Scopus. These databases were searched from inception through April 2018. Using the predefined literature review methodology, 347 articles were identified for analysis, with the earliest dating back to 1903 [7]. From these 347 articles, articles were further screened for this proof-of-concept study to focus on articles that made an assertion, or claim, about the annual rate of US physicians who die of suicide. Then, 1 author (TIL) established a Google Scholar alert using the keyword “physician suicide” and screened additional articles from peer-reviewed journals to include based on earlier established inclusion criteria from the published scoping literature review [7]. These articles, published through March 2020**,** were identified and added to the article set used for this study.

Websites, news articles, blogs, white papers, organizational or institutional reports, and other gray literature were not the primary focus of this study and therefore not retrieved for inclusion as original sources of the annual suicide rate claim. However, additional articles, websites, or other links were only added to the set of claims if they were cited by a peer-reviewed article already included in the data set.

### Data Extraction

Manual data extraction was performed by 1 author (TIL) to collect article (or resource) type, title, authors, digital object identifier or HTTP URI, publication year, claim (about annual physician suicide rate), data of last access of the article (eg, for a webpage), and the citations that the authors indicated supported the claim. Data were extracted into a spreadsheet that was then used to create nanopublications. The spreadsheet also notes the original sources of the reference for the set, which are as follows: scoping literature review [7], Google Scholar alert, or not applicable as the citation is included because it is referenced by another claim.

To ensure that the citations provided to support a claim were sufficiently identified, the sentence preceding and following the claim of interest was checked for a citation. Table 1 illustrates an example of the extraction procedure on the level of the manuscript and claim.

**Table 1:** Claim identification and attribution during data extraction.

Claim of interest, including preceding and following sentences	Claim extracted	Citations to which the claim is attributed
“There is an urgent need for development and dissemination of these best practices. An estimated 300 physicians die by suicide per year, and rates may be rising.^43,44^ Each time, the headlines are saddening—even shocking” [12].	“An estimated 300 physicians die by suicide per year, and rates may be rising” [12].	Apropos claim citation No. 43: Facts about physician depression and suicide [13] Apropos claim citation No. 44: Physician Burnout and Well-Being: A Systematic Review and Framework for Action [14]
“And the mortality is high. In male doctors the suicide risk is 1.4 times that of the general population and for female doctors it is an astounding 2.27. We know that 300–400 physicians commit suicide every year. And there likely are more, since some death certificates may not reflect the actual cause of death” [15].	“We know that 300–400 physicians commit suicide every year” [15].	No citation provided

For websites, a version of the website with a last access date was retrieved for data extraction using Internet Archive’s Wayback Machine [16]. If a claim was available, then this text was extracted as the claim; if none, then the nanopublication included the comment “No apparent claim of annual physician suicide rate”; if no archived version of the website was available, then the nanopublication included the comment “Unverified claim of annual physician suicide rate present.” A separate nanopublication was created for each different cited version of a website if it was cited at 2 different time points by different articles.

### Data Structure

Each nanopublication consists of 3 components: assertion, provenance, and publication information [17]. Following the nanopublication model of Groth et al [1], the steps taken to create a nanopublication for each claim about physician suicide incidence involved the following:

1. Assertion: represented as a set of triples—the subject is the local article or resource identifier, which is linked via creator, date, identifier, title, type, citation, and comment.
2. Provenance: each assertion is linked to the creator (annotator), who is identifiable by an Open Researcher and Contributor Identifier account.
3. Publication information: each nanopublication contains a time stamp, the creator, link to the template, and public key plus signature.

We created a literature-based claim template to specify these fields and values and provide mappings to semantic types and relations using Resource Description Framework Schema, Nanopublication ontology, the Fabio ontology for document types, the Provenance, Authoring and Versioning ontology for provenance, and the Semanticscience Integration Ontology for citations.

### Creating Nanopublications

**Figure 1:**
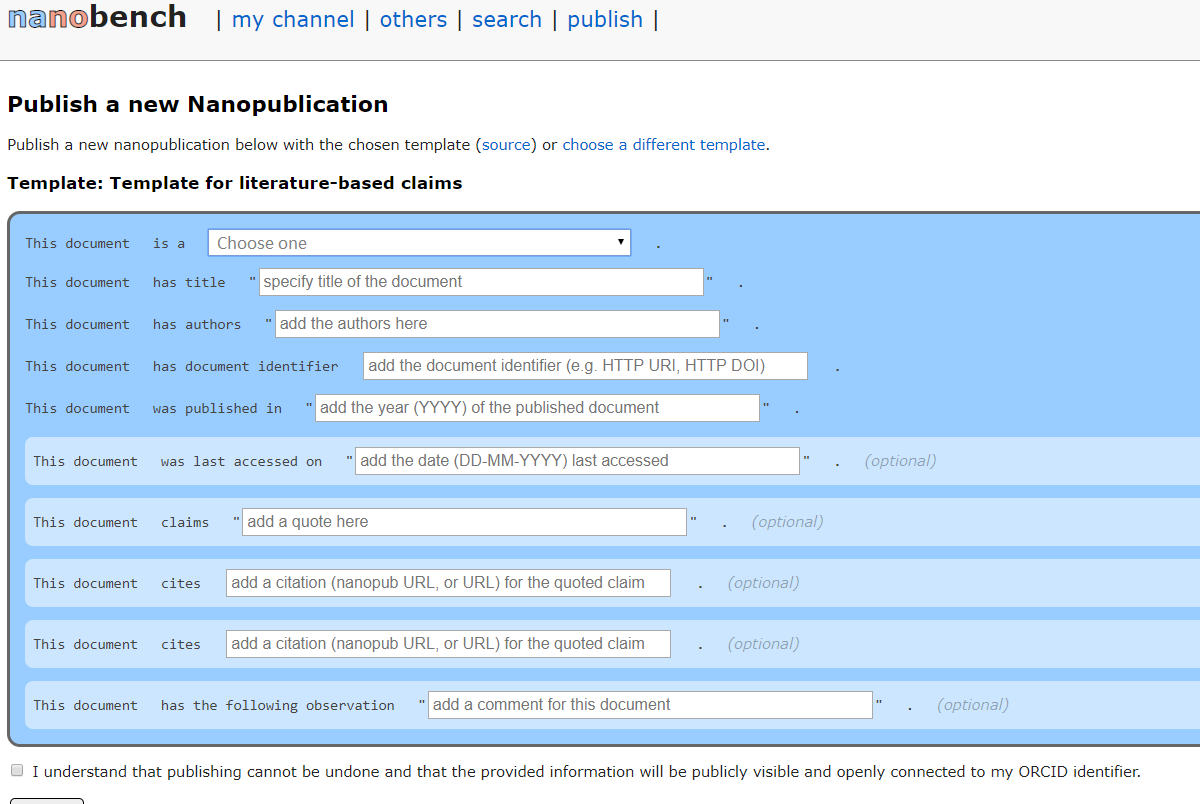
Nanobench template for literature-based claims. DOI: Digital Object Identifier; ORCID: Open Researcher and Contributor Identifier.

A nanopublication was created for each article or resource using *Nanobench* with the literature-based claim nanopublication template (Figure 1) [18]. *Nanobench* is a Java based end-user tool that allows for browsing and publishing of nanopublications. By connecting to the decentralized nanopublication network [19], users can see other people’s nanopublications and publish their own via forms generated from specific templates, which are themselves defined and published as nanopublications. All published nanopublications are digitally signed and linked to the user’s Open Researcher and Contributor Identifier account [20]. A nanopublication index was then created containing all created nanopublications.

### Ethical Considerations

The study involved data derived from resources published and available in the public domain. No institutional review board approval was required.

## Results

**Figure 2:**
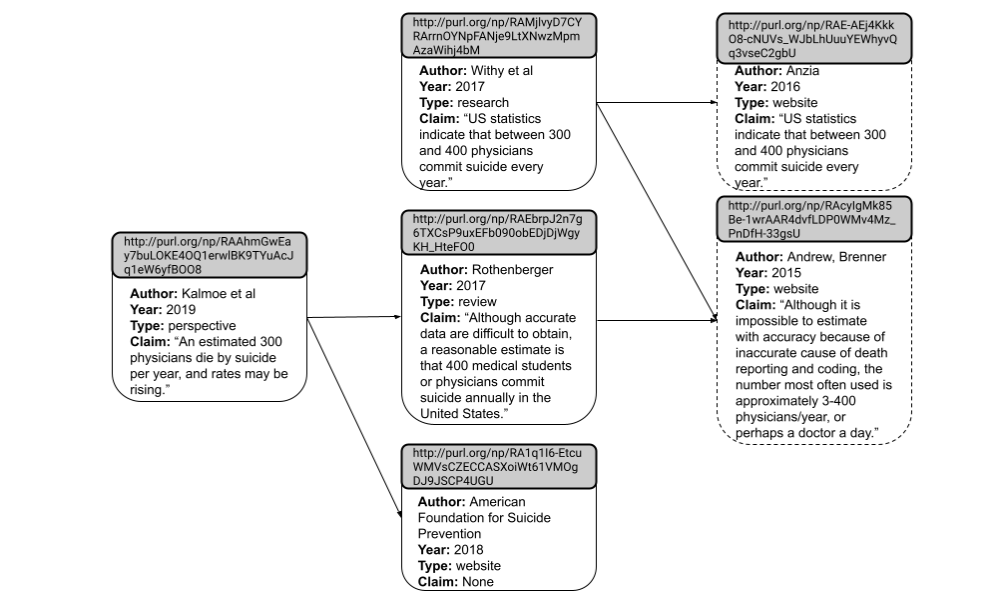
Nanopublications linked by their claims, if made, and nanopublications cited as source of the claim, if available. Nanopublications appearing in light gray with dashed lines represent an article or resource that states a claim about annual physician suicide rate but provides no further references.

A set of 49 claims concerning the rate of US physician suicide was represented as nanopublications. Figure 2 [12-14,21,22] illustrates 1 published peer-reviewed article [12] represented as a nanopublication, which was then linked to its citation for the claim, which was also represented as a nanopublication along with the associated chain of nanopublished claims. For example, Kalmoe et al [12] claimed in a 2019 perspective article, “An estimated 300 physicians die by suicide per year, and rates may be rising.” This claim was accompanied by citations of 2 resources, a 2018 version of a website from the American Foundation for Suicide Prevention (AFSP) [13] and a literature review published in peer-reviewed literature [14]. Each of these resources was reviewed to extract information to create nanopublications. The AFSP website did not make this claim. Rothenberger et al claimed in 2017 that “Although accurate data are difficult to obtain, a reasonable estimate is that 400 medical students or physicians commit suicide annually in the United States.” This claim was accompanied by the citation of a 2015 version of a website from Medscape, a medical news platform, which claimed “Although it is impossible to estimate with accuracy because of inaccurate cause of death reporting and coding, the number most often used is approximately 3-400 physicians/year, or perhaps a doctor a day” [23]. This website stated a claim about annual physician suicide rate but provided no further references. Withy et al claimed in 2017 that “US statistics indicate that between 300 and 400 physicians commit suicide every year” [21] and also cited the same Medscape website [23], as well as a website from StatNews, another medical news platform, which claimed “US statistics indicate that between 300 and 400 physicians commit suicide every year” [22]. This website also stated a claim about annual physician suicide rate but provided no further references. As a result, this claim network ends, as demonstrated in Figure 2.

**Figure 3:**
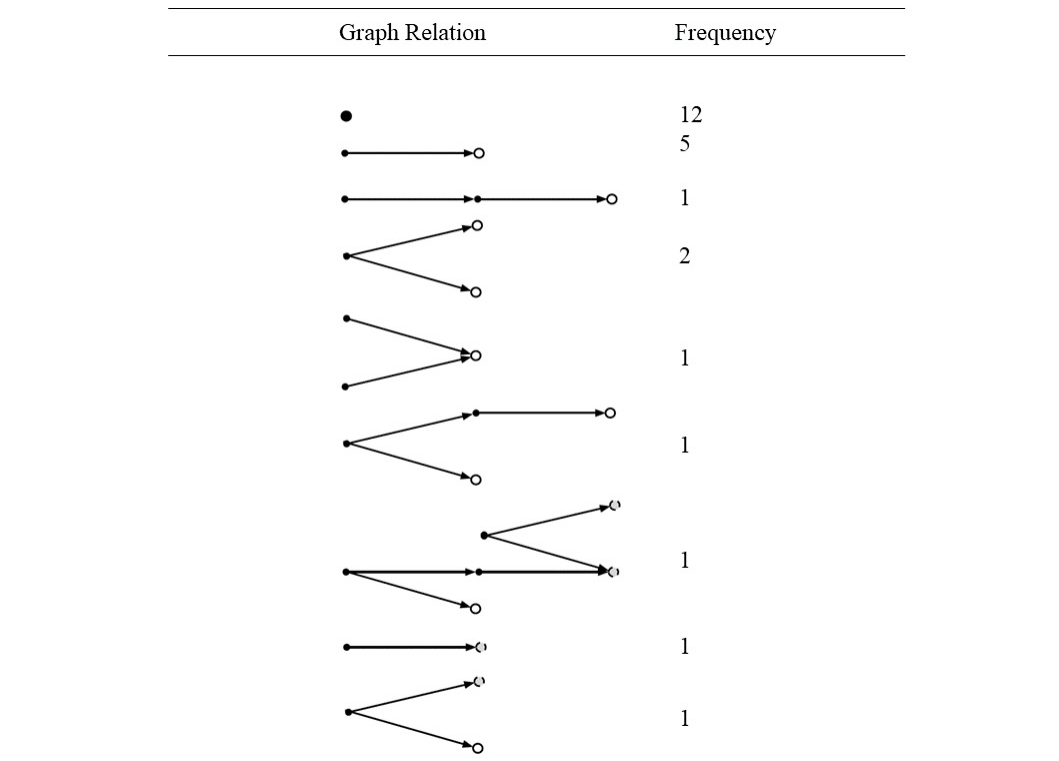
Multiple graphs linking nanopublications representing US physician suicide rate claims. Closed circles: the nanopublication represents an article that states a claim about annual physician suicide rate. Open circles: the nanopublication represents an article or resource that has “No apparent claim of annual physician suicide rate” or it is “Unverified if claim of annual physician suicide rate present.” Gray circles: the nanopublication represents an article or resource that states a claim about annual physician suicide rate but provides no further references.

Nanopublications and claim networks were created for all included articles in the data set, similar to how Figure 2 was created (Figure 3). Figure 3 shows multiple claim networks created, without a single primary source or end point for linked nanopublications. This resulted in 12 stand-alone nanopublications unlinked to any others because of the absence of a citation to support the asserted claim. An additional 8 types of graphs were created, where all nanopublications resulted in one of two possible end points: (1) no apparent claim of annual physician suicide rate was identified, or it was unverified if claim of annual physician suicide rate is present; (2) a claim about annual physician suicide rate was asserted, but no further citations are provided to support the claim.

Although not an a priori objective of this study, applying the nanopublication schema to annual physician suicide claims enabled the capture of variant claims published on a website. Specifically, the website for the AFSP was cited 6 times between 2011 and 2018. Surprisingly, while only the 2018 version of the AFSP website could be retrieved, it contained no apparent claim of annual physician suicide rate. It could not be determined whether a previous version of the website may have stated the claim but then was subsequently removed.

In another instance, the physician suicide claim appeared to have changed over time; the Medscape website was cited by articles as a 2015 and 2018 version, each represented by different nanopublications. The 2015 version of the Medscape website retrieved from Internet Archive’s Wayback Machine stated the following: “It has been reliably estimated that on average the United States loses as many as 400 physicians to suicide each year (the equivalent of at least one entire medical school).” However, the 2018 version of the website, which used the same link, stated the claim differently, which is as follows: “Although it is impossible to estimate with accuracy because of inaccurate cause of death reporting and coding, the number most often used is approximately 3-400 physicians/year, or perhaps a doctor a day.”

## Discussion

### Principal Results

The findings of this study emphasize the importance of integrating scientific literature, especially individual scientific claims, in a reliable and verifiable manner. Creating nanopublications to represent articles’ claims that “300 to 400 U.S. physicians die by suicide annually” empirically demonstrates that this is a poorly supported yet frequently stated claim. No single source of the US physician suicide rate claim studied could be identified.

This study is the first known application of nanopublication infrastructure to scientific claims regarding physician suicide. Applying the nanopublications schema to physician suicide claims revealed that (1) the claim network is not fully connected, (2) no single primary source of the claim is available, and (3) all end-point citations had a claim with no further citation, had no apparent claim, or could not be accessed to verify the claim. This is in line with previous findings [8] and expands on existing work by representing physician suicide rate claims as nanopublications. Representing these claims as nanopublications can be extended and improved. This study has important implications.

Because the use of nanopublications has not been applied to suicidology research, in particular physician suicide, this proof-of-concept study may highlight the need for more coordinated research efforts in these fields. Some biomedical research communities have already benefited from such an approach to their research efforts. It is important to note that to achieve this goal, a minimum set of community agreed-upon annotations would be needed to optimize nanopublication quality [1]. In the study of physician suicide, no such community standards exist yet, but this could also be an opportunity to develop such standards, driving the application of nanopublication in this field from the ground up. Such further work could address an imperative that has previously been identified in the study of physician suicide incidence [7].

Nanopublications can allow for continued claim tracing and verification, including, for example, accounting for versioning. Different website versions may even differ in their assertions of the claim, which was identified in this study even though it was not a stated aim of the study. This study builds on previous work by applying nanopublication infrastructure to the articles and claims they make. As earlier noted, the use of nanopublications to represent scientific assertions has been conducted for the genetic basis for disease pathophysiology [3]; Alzheimer disease research [5]; and additional life sciences data, including data on diseases, genes, proteins, drugs, biological pathways [6].

### Limitations

One limitation is that the claim network contains only verbatim claims about the annual physician suicide rate. The first study estimating incidence from 2 years of obituary data from a medical professional organization was published in 1968, reporting a crude annual suicide rate of 38.4 per 100,000 physicians [24]. Since then, systematic reviews or meta-analyses have sought to aggregate data from other observational studies estimating incidence [25-28]. Most studies about suicide incidence should report a suicide mortality rate, which is the number of deaths by suicide per 100,000 person-years, and physician suicide mortality rates have yet to be nanopublished. Further work is needed to represent all available data on physician suicide, beyond focusing on the single claim studied here. Representing additional data as nanopublications, including incidence data, risk factors, demographics, and other contextual information, may offer an even richer graph of existing knowledge about physician suicide to enable more rapid learning about the field.

Second, regarding the data source approach, snowballing to examine full reference lists of included articles was not performed. The focus for this proof-of-concept study was to specifically focus on the citation that the author of an article provides at the end of the sentence that makes the annual suicide rate claim. Snowballing may reveal additional publications that make the same claim, but we also anticipate that this approach would add further evidence that the claim network about annual suicide rate would reveal addition fragmented and disconnected parts of the network. Additional investigation would be needed to explore this hypothesis.

Moreover, the geographical focus of the claims in this study is in the United States, although physician suicide is a global issue. Dutheil et al [28] conducted a meta-analysis that included peer-reviewed literature from North America, Europe, Africa, Australia, and Asia. The literature review that served as a data source for this study also identified 37 countries where physician suicide was reported [7]. Incorporating country of origin and death by suicide, when available, into nanopublications about physician suicide could further enrich understanding physician suicide in a global context.

Finally, there may be a limitation based upon the search strategy that contributed to the data source used for this study. As web search may also offer a valuable source of nonpeer-reviewed literature and gray literature that also make a claim similar to “300 to 400 U.S. physicians die by suicide annually,” these may offer an unstudied area of misinformation in public-facing publications about physician suicide. As this study was not designed as an infodemiology study, however, incorporating such a search to enrich the data source and further analysis could add to the current literature about physician suicide.

### Conclusions

Nanopublications remain to be adopted in broader scientific publishing in medicine, and especially in publishing about physician mental health and suicide. Our work integrates these various claims and enables the verification of nonauthoritative assertions, thereby better equipping researchers to advance evidence-based knowledge and make informed statements in the advocacy of physician suicide prevention.

## Abbreviations

AFSP: American Foundation for Suicide Prevention

## Data Availability

All data are openly available. Manually extracted data from each of the claims were stored in a spreadsheet [29], then subsequently published as individual nanopublications, and aggregated into a nanopublication index [30]. *Nanobench* is available for use without licensing fees [20].
